# Acute and Post-acute Neuromodulation Induces Stroke Recovery by Promoting Survival Signaling, Neurogenesis, and Pyramidal Tract Plasticity

**DOI:** 10.3389/fncel.2019.00144

**Published:** 2019-04-12

**Authors:** Ahmet B. Caglayan, Mustafa C. Beker, Berrak Caglayan, Esra Yalcin, Aysun Caglayan, Burak Yulug, Lutfu Hanoglu, Selim Kutlu, Thorsten R. Doeppner, Dirk M. Hermann, Ertugrul Kilic

**Affiliations:** ^1^Department of Physiology, Faculty of Medicine, Istanbul Medipol University, Istanbul, Turkey; ^2^Regenerative and Restorative Medical Research Center, Istanbul, Turkey; ^3^Department of Medical Biology, Faculty of Medicine, Istanbul Medipol University, Istanbul, Turkey; ^4^Department of Neurology, Faculty of Medicine, Alanya Alaaddin Keykubat University, Antalya, Turkey; ^5^Department of Neurology, Faculty of Medicine, Istanbul Medipol University, Istanbul, Turkey; ^6^Department of Physiology, Faculty of Medicine, Necmettin Erbakan University, Konya, Turkey; ^7^Department of Neurology, Faculty of Medicine, University of Goettingen, Göttingen, Germany; ^8^Department of Neurology, Faculty of Medicine, University of Duisburg-Essen, Essen, Germany

**Keywords:** neurodegeneration, apoptosis, tissue remodeling, rTMS, cerebral ischemia

## Abstract

Repetitive transcranial magnetic stimulation (rTMS) has gained interest as a non-invasive treatment for stroke based on the data promoting its effects on functional recovery. However, the exact action mechanisms by which the rTMS exert beneficial effects in cellular and molecular aspect are largely unknown. To elucidate the effects of high- and low-frequency rTMS in the acute-ischemic brain, we examined how rTMS influences injury development, cerebral blood flow (CBF), DNA fragmentation, neuronal survival, pro- and anti-apoptotic protein activations after 30 and 90 min of focal cerebral ischemia. In addition, inflammation, angiogenesis, growth factors and axonal outgrowth related gene expressions, were analyzed. Furthermore, we have investigated the effects of rTMS on post-acute ischemic brain, particularly on spontaneous locomotor activity, perilesional tissue remodeling, axonal sprouting of corticobulbar tracts, glial scar formation and cell proliferation, in which rTMS was applied starting 3 days after the stroke onset for 28 days. In the high-frequency rTMS received animals reduced DNA fragmentation, infarct volume and improved CBF were observed, which were associated with increased Bcl-xL activity and reduced Bax, caspase-1, and caspase-3 activations. Moreover, increased angiogenesis, growth factors; and reduced inflammation and axonal sprouting related gene expressions were observed. These results correlated with reduced microglial activation, neuronal degeneration, glial scar formation and improved functional recovery, tissue remodeling, contralesional pyramidal tract plasticity and neurogenesis in the subacute rTMS treated animals. Overall, we propose that high-frequency rTMS in stroke patients can be used to promote functional recovery by inducing the endogenous repair and recovery mechanisms of the brain.

## Introduction

Ischemic- stroke is a global health-care condition which mainly results in motor impairment and causes long-term disability (Langhorne et al., 2009). Brain injury following ischemic-stroke develops from free radical generations, excitotoxicity, peri-infarct depolarizations, inflammation, and apoptosis (Dirnagl et al., 1999). While brain has endogenous capacity to protect neurons and restore the lost neuronal function, this capacity is often limited and insufficient to promote survival and recovery. Therefore, both protection of neurons in the acute-stroke and promotion of post-stroke motor recovery are believed to be critical in stroke treatment. Motor performance is also affected from the imbalanced interhemispheric inhibition which results in the increased inhibition of the ipsilateral primary motor cortex and hence, decreased motor outcome (Liepert et al., 2000; Murase et al., 2004).

Transcranial magnetic stimulation (TMS), a non-invasive neuromodulation technique of alternating magnetic fields, has been proposed to enhance cortical excitability in the affected hemisphere (Khedr et al., 2005; Lefaucheur, 2006). Recently, repetitive TMS (rTMS) which uses short and repeated magnetic bursts has been demonstrated to exert longer lasting effects than non-repetitive TMS (Hsu et al., 2012; Chervyakov et al., 2015) and is currently being investigated for the treatment of several neurological and psychiatric conditions including depression (Dell’osso et al., 2011), Parkinson’s disease (Wagle Shukla et al., 2016), Alzheimer’s disease (Koch et al., 2017), and stroke (Lefaucheur, 2006).

Recent experimental studies revealed that 60 Hz TMS treatment attenuates tail and limp paralysis, oxidative stress and cell death via activation of antioxidant system after experimental autoimmune encephalomyelitis in rats (Medina-Fernandez et al., 2017). It was revealed that high frequency rTMS increases cell proliferation and synaptic plasticity and inhibits apoptotic cell death through activation of BDNF, CREB, ERK, and AKT signaling pathways in neuronal cells after oxygen glucose deprivation *in vitro* (Baek et al., 2018). Furthermore, it was shown that rTMS ameliorates cognitive deficits, lesion size via activation of Bcl-2 and inhibition of Bax after ischemic stroke (Guo et al., 2017) and cytochrome-c release and inflammation after hemicerebellectomy in rats (Sasso et al., 2016). Although rTMS has gained great interest in treatment of patients with neurodegenerative disorders, there are inconsistent data on the efficacy of high and low-frequency rTMS in cellular and molecular aspects.

Although there are several studies conducted in humans, the action mechanism responsible for the positive effects of rTMS treatment has not been fully elucidated, partially due to the lack of detailed experimental studies. In this context, we conducted these studies to investigate the molecular and physiological changes induced by inhibitory low (1 Hz) and excitatory high-frequency rTMS (20 Hz) on the acute pathophysiological events and sub-acute recovery processes after occlusion of the middle cerebral artery (MCA) in mice. Here, we provide evidence that high-frequency rTMS induces neuronal survival and regional cerebral blood flow (CBF), while reducing infarct volume and apoptotic cell death. Furthermore, high-frequency rTMS also induced neurogenesis, neuronal plasticity, perilesional tissue remodeling, promoted axonal sprouting and subsequent functional recovery as well as changes in the levels of inflammation-, growth-, angiogenesis-, or neuronal plasticity- related genes in mice.

## Materials and Methods

### Animals and Experimental Procedures

**FIGURE 1:**
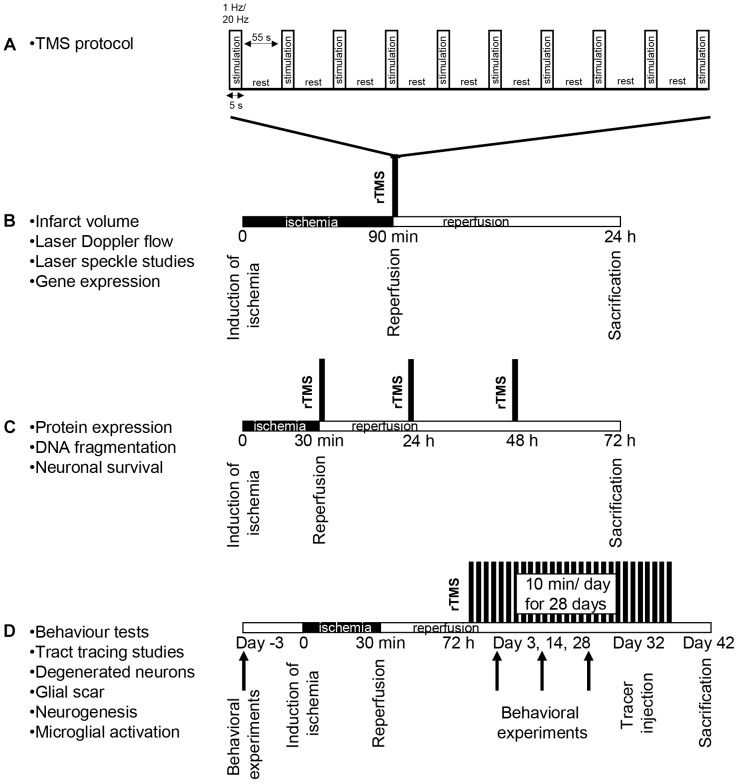
Experimental design. Stimulation method of repetitive transcranial magnetic stimulation (rTMS) **(A)** and experimental protocol for 90 min ischemia 24 h reperfusion set **(B)**, for 30 min ischemia 72 h reperfusion set **(C)** and for long term set following 30 min ischemia **(D)**. In the first experimental setup, animals received either low-frequency (1 Hz) or high-frequency (20 Hz) rTMS for a total of 10 min with repeated 5 s stimulation and 55 s resting periods with the onset of reperfusion **(A)**. Control animals were placed under the rTMS coil but not subjected to stimulation. All rTMS stimulations were carried out under anesthesia and control animals received sham stimulation under anesthesia.

All experimental procedures were carried out with government approval according to NIH guidelines for the care and use of laboratory animals. Ethical committee approval was obtained from Istanbul Medipol University. In this study, three sets of experiments were performed to examine the effects of rTMS stimulation (Figure 1A) on the short term and long term outcomes of ischemic stroke; (i) for the analysis of infarct volume- (*n* = 8–9), laser speckle imaging- (LSI) and gene expression- analyses (*n* = 5) (Figure 1B); (ii) for the analysis of disseminate ischemic injury, protein expression, DNA fragmentation, and neuronal survival (Figure 1C; *n* = 6–7 mice/group), and (iii) for the analysis of long-term behavioral analyzes, tract tracing studies, neuronal degeneration, glial scar formation, microglial activation and cell proliferation (Figure 1D; *n* = 12 mice/group). Experimental protocols were summarized in Figure 1. In brief, each experimental set contained 10–12 week-old male Balb/c mice (22–25 g). In the first set, animals were subjected to 90 min of focal cerebral ischemia (FCI), followed by 24 h reperfusion, in this model necrotic cell death following infarct formation is observed (Figure 1B). In the second, animals were subjected to 30 min ischemia, followed by 72 h reperfusion induces disseminated or apoptotic cell death (Figure 1C). In the third set, animals were subjected to 30 min ischemia, followed by 42 days reperfusion, it is a short term ischemia and allows animal survival for a long term experiment in the context of ethical concerns (Figure 1D). Animals were maintained under a constant 12:12-h light–dark regimen with ad libitum access to food and water. In the each set, animals were divided into three groups based on the application regimen of repetitive transcranial magnetic stimulation (rTMS); control group (did not receive rTMS), 1 Hz group (received 1 Hz rTMS) and 20 Hz group (received 20 Hz rTMS) under 1% isofluorane anesthesia.

### Induction of Focal Cerebral Ischemia

Focal cerebral ischemia was induced by middle cerebral artery occlusion (MCAo). Animals were anesthetized with 1% isofluorane (30% O_2_, reminder N_2_O), and rectal temperature was maintained between 36.5 and 37.0°C using a feedback-controlled heating system. During the experiments, CBF was measured using a laser Doppler flowmetry (LDF) with a flexible 0.5 mm fiber optic probe (Perimed), which was attached to the intact skull overlying the MCA territory (2 mm posterior/6 mm lateral from bregma). LDF changes were monitored up to 1 min after the onset of reperfusion or just before rTMS treatment. For the induction of FCI, a midline neck incision was made, and the left common and external carotid arteries were isolated and ligated. A microvascular clip (FE691; Aesculap) was temporarily placed on the internal carotid artery. A 8-0 nylon monofilament (Ethilon; Ethicon) coated with silicon resin (Xantopren; diameter of the coated thread: 180–190 μm) was introduced through a small incision into the common carotid artery and advanced 9 mm distal to the carotid bifurcation for MCAo. In the first set in which mice were exposed to 90 min of MCAo, animals were sacrificed under deep anesthesia (4% isofluorane) 24 h after the onset of MCAo. Brains were removed, frozen on dry ice, and cut on a cryostat into coronal 18-μm sections for studying infarct volume. In the second set, animals were sacrificed 72 h after the onset of MCAo and in the third set, animals were sacrificed 42 days after MCAo. In the third set, to label proliferating cells, 50 mg/kg 5-Bromo-2′-deoxyuridine (BrdU, #B5002 Sigma) was intraperitoneally injected to all animals starting from day 3 to the end of the experiment after MCAo (Doeppner et al., 2017) and animals were transcardially perfused with 0.9% NaCl at 42 days after the MCAo. Brain tissue was frozen on dry ice and cut on a cryostat into 18-μm coronal sections (Kilic et al., 2010).

### Repetitive Transcranial Magnetic (rTMS) Stimulation

To examine the effects of rTMS stimulation on the short term and long term outcomes of ischemic stroke, three sets of experiments were carried out. In the each set, animals were divided into three groups based on the application regimen of rTMS; control group (did not receive rTMS), 1 Hz group and 20 Hz group. In the first set, mice were exposed to 90 min of MCAo, received one session of stimulation for 10 min under anesthesia immediately after the induction of reperfusion. In the second set, mice were exposed to 30 min of MCAo and received three sessions of stimulation for 10 min per day under anesthesia starting from the induction of reperfusion. In the third set, animals were subjected to 30 min MCAo. A regimen of 10 min rTMS per day for 28 days was initiated 3 days after the ischemia/reperfusion. Each 10 min treatment session consists of 5 s stimulation followed by 55 s rest period repeated for 10 times and performed under anesthesia. Control animals were placed under the rTMS coil under anesthesia but did not receive stimulation.

In all sets, a magnetic stimulator (Neurosoft, Avm Saglik) with a figure-of-eight coil (38 mm inner diameter, 94 mm outer diameter) was used to stimulate rTMS. Although both hemispheres were affected by rTMS stimulation to some extend, the stimulation site was set as the primary motor cortex (left M1) by placing the rTMS device over. Motor evoked potentials (MEPs) were measured at the biceps femoris (BF) muscle of the right hind limb using an electromyography as previously described (Ogiue-Ikeda et al., 2003; Yoon et al., 2011). The resting motor threshold (RMT) was defined as the lowest stimulator output where the peak-to-peak amplitude of MEP was greater than 5% of its maximal amplitude in at least half of 10 trials. The rTMS stimulation was applied as follows; 5 s stimulation followed by 55 s rest for a total duration of 10 min at either 1 Hz or 20 Hz. Stimulation intensity was set at 100% of the RMT (i.e., 26% of the maximum output of the stimulator).

### Infarct Volume Analysis

For the evaluation of infarct volume, coronal brain sections were collected at four equidistant brain levels, 2 mm apart, from mice exposed to 90 min MCAo, which were stained with cresyl violet according to a standard protocol (Caglayan et al., 2017). For each section, the border between infarcted and non-infarcted areas was outlined using ImageJ (National Institute of Health, Bethesda, MD, United States). Infarct area was calculated by subtracting the area of the non-infarcted ipsilateral hemisphere from that of the contralateral side. Infarct volume was calculated by integration of infarct areas.

### Laser Speckle Imaging (LSI)

To evaluate the effect of rTMS on microcirculation, LSI was carried out as described previously (Beker et al., 2015) with minor modifications. Briefly, mice were exposed to 90 min MCAo as described above. Starting 1 min after reperfusion, real-time CBF changes were recorded by Pericam PSI System (Perimed). To evaluate the CBF changes in the ischemic core and ischemic penumbra regions of interest (ROI) covering 1.0 mm × 5.5 mm (in lateral and rostrocaudal direction, respectively) were defined 0.5 and 1.5 lateral and 0.5 mm posterior to the bregma, in which mean CBF was calculated using a blood perfusion imaging software (PIMSoft; Perimed). Regional CBF was recorded throughout the 60 min observation period after rTMS treatment. From the measurements obtained, relative CBF changes (in %) at the end of the observation period which is compared with the values of before rTMS treatment after MCAo.

### Analysis of DNA Fragmentation/Apoptosis

For the evaluation of neuronal injury, coronal brain sections at the level of the bregma of mice exposed to 30 min MCAo were fixed with 4% paraformaldehyde (PFA)/0.1 M phosphate buffered saline (PBS) and were labeled using a TUNEL kit (In Situ Cell Death Detection Kit; Roche). Sections were counterstained with 4′, 6-diamidino- 2-phenylindole (DAPI). Stainings were analyzed by quantifying DNA fragmented cells (which in 30 min MCAo are equivalent to neurons) in 12 adjacent ROI in the striatum, each measuring 62,500 μm^2^, under a laser scanning confocal microscope (LSM 780, Carl Zeiss) (Caglayan et al., 2017).

### Analysis of Neuronal Survival

For the analyzes of neuronal survival, adjacent brain sections of the same animals were fixed in 4% paraformaldehyde (PFA)/0.1 M PBS, washed and immersed for 1 h in 0.1 M PBS containing 0.3% Triton X-100 (PBS-T)/10% normal goat serum. Sections were incubated overnight at 4°C with Cy3-conjugated monoclonal mouse anti-NeuN (MAB377C3; Chemicon). Next day, sections were incubated with 4′, 6- diamidino-2-phenylindole (DAPI). Sections were analyzed using a laser scanning confocal Zeiss LSM780 microscope (Carl Zeiss). Nine different ROI in the striatum, each measuring 62,500 μm^2^, were evaluated. Mean numbers of NeuN+ cells were analyzed in the ischemic and contralesional striatum. By dividing results obtained in both hemispheres, the percentage of surviving neurons in the ischemic striatum was determined.

### Analysis of Capillary Density

For the analysis of capillary density, brain sections of the same animals were fixed in 4% PFA/0.1 M PBS, washed and immersed for 1 h in 0.1 M PBS containing 0.3% Triton X-100 (PBS-T)/10% normal goat serum. Sections were incubated overnight at 4°C with anti-CD31 (ab28364; Abcam) that were detected with Alexa Fluor 488-conjugated secondary antibody. Next day, sections were washed with 0.1 M PBS and incubated with DAPI. Sections were evaluated by counting the number of CD31 positive vessel profiles in rectangular fields, measuring 500,000 μm^2^.

### Western Blot Analysis

Tissue samples from ischemic striatum of control (no treatment), 1 Hz rTMS or 20 Hz rTMS applied animals were obtained 72 h after 30 min of MCAo. To analyze the apoptotic protein expressions, Western blot of these samples were performed as previously described (Kelestemur et al., 2016; Beker et al., 2017). Membranes were blocked with 5% non-fat milk (Blotto, #sc-2324, ChemCruz) in Tris-buffered saline (TBS) containing 0.1% Tween (TBS-T; blocking solution) for 1 h at room temperature, washed in TBS-T, and incubated overnight with a primary antibody against Bcl-xL (#2764, Cell Signaling), Bax (#2772, Cell Signaling), cleaved caspase-1 (#22165, Santa Cruz), total caspase-3 (ab13847, Abcam), or cleaved caspase-3 (#9664, Cell Signaling). Individual blots were performed at least three times. Protein loading was controlled by stripping and reprobing with anti-β-actin antibody (#4970, Cell Signaling). Protein levels were analyzed densitometrically using an image analysis software (ImageJ; National Institute of Health, Bethesda, MD, United States), corrected with values determined on β-actin blots and expressed as relative values compared with the control group.

### Gene Expression Analysis by RT-PCR

Tissue samples from striatum of control (no treatment), 1 Hz rTMS or 20 Hz rTMS applied animals were obtained 24 h after 90 min of MCAo. Total RNA was extracted using AllPrep DNA/RNA/Protein Mini Kit (#80004, Qiagen). Quality and quantity of RNA samples were determined by spectrophotometric analysis. cDNA was synthesized from 1 μL total RNA using Transcriptor First Strand cDNA Synthesis Kit (#04896866001, Roche). qPCR was performed in triplicates for each sample and repeated three times using SsoAdvanced Universal SYBR Green Supermix (#172-5272, Bio-Rad) in CFX Connect^TM^ Real-Time PCR Detection System (#185-5201, Bio-Rad). GAPDH and β-actin were used as internal controls. Relative expression of each gene was calculated as fold-change of the control using the comparative Ct method (2^-ΔΔCt^) and normalized to GAPDH and β-actin. Primer pairs used are given in Supplementary Table S1.

### Functional Neurological Tests

#### Grip Strength Test

The grip strength test consists of a spring balance coupled with a Newtonmeter (Medio-Line Spring Scale, metric, 300 g) that is attached to a triangular steel wire, which the animal instinctively grasps. When pulled by the tail, the animal exerts force on the steel wire (Kilic et al., 2010). Grip strength was evaluated at the right paretic forepaw, the left non-paretic forepaw being wrapped with adhesive tape. Grip strength was evaluated five times on occasion of each test, for which mean values were calculated. From these data, percentage values (post-ischemic vs. pre-ischemic) were computed. Pre-ischemic results did not differ between groups.

#### RotaRod Test

RotaRod test was used for the analyses of the effect of rTMS on motor coordination. The RotaRod is a rotating drum with a speed accelerating from 6 to 40 rpm (model 47600;Ugo Basile, Comerío, Italy),which allows to assess motor coordination skills (Kilic et al., 2014). Maximum speed is reached after 245 s, and the time at which the animal drops off the drum is evaluated (maximum testing time: 300 s). Measurements were performed five times each on the same occasion when grip strength was evaluated. For all five measurements, mean values were computed, from which percentage values (post-ischemic vs. pre-ischemic) were calculated. Pre-ischemic data did not differ between groups.

#### Open Field Test

Open field test was used for the evaluation of locomotor activity of animals. The open field is a round arena (diameter: 150 cm) covered by a white plastic floor, surrounded by a 35 cm high sidewall made of white polypropylene, which allows to measure spontaneous locomotor activity and exploration behavior (Kilic et al., 2008). The arena is divided into three sections, including an outer wall zone (17.7% of diameter, close to the wall), an intermediate transition zone (32.3% of diameter), and an inner zone (50% of diameter, the center of the arena). Each mouse was released near the wall and observed for 10 min. Animal paths were tracked with an electronic imaging system (Anymaze, Stoelting Europe). To determine measures of exploratory behavior and anxiety, the time resting and progressing were assessed.

#### Tail Suspension Test

For the analysis of depressive-like state of animals, tail suspension test was performed in mice that were suspended 50 cm above the floor using an adhesive tape placed 1 cm from the tip of the tail. Total immobility time was recorded for 5 min, indicating depressive-like state of animals.

### Assessment of Brain Atrophy, Degeneration, and Glial Scar

For the evaluation of striatum atrophy, coronal brain sections were taken from the animals of the third set, stained with cresyl violet according to a previously described protocol (Kilic et al., 2014). Striatal area was outlined using an image analysis system (Image J; National Institutes of Health). For the evaluation of degenerating cells, sections were stained with Fluorojade C (#AG325 Millipore) according to manufacturer’s protocol. In case of GFAP stainings, the area of scar tissue was outlined using the Zen Blue software (version 2012; Carl Zeiss).

### Immunohistochemistry for Cell Proliferation Studies and Microglial Activation

To analyze the proliferating cells, 18-μm thick brain sections were prepared using a cryostat (CM1950, Leica) from the animals of the second set. Sections were then blocked in 10% goat serum (#G9023, Sigma) in PBS solution and incubated with monoclonal rat anti-BrdU antibody (#ab6326, Abcam). To identify the BrdU(+) proliferating cells, double staining was performed using anti-NeuN (#MAB377C3, Millipore), anti-GFAP (#3656, Cell Signaling) or anti-Iba1 (#019-1974, Wako) antibodies overnight at 4°C. On the next day, sections were washed with PBS and incubated with the appropriate secondary antibodies for 1 h at room temperature. Sections were then imaged with a laser scanning confocal microscopy (LSM 780, Carl Zeiss). Nine different ROI in the ischemic striatum, each measuring 62,500 μm^2^, were evaluated. Numbers of double immunopositive cells were counted blindly.

### Delivery of Biotinylated Dextran Amine (BDA) and Axonal Projection Analysis

To evaluate the axonal projections induced by rTMS treatment, animals were subjected to 30 min of MCAo and received no treatment, 1 Hz rTMS or 20 Hz rTMS for 28 days. At the end of the completion of behavior tests, 10% Biotinylated Dextran Amine (BDA, 10,000 MW, #D1956, Invitrogen) diluted in 0.01 M PBS was injected into contralateral motor cortex (0.5 mm rostral and 2.5 mm lateral to the bregma) of animals as described previously (Kilic et al., 2014). Sections were washed in PBS, blocked with 10% normal goat serum in PBS for 1 h at room temperature and incubated with AlexaFluor 555 conjugated streptavidin (#S21381, Invitrogen) for 90 min at room temperature. Sections were then imaged with confocal microscopy (LSM 780, Carl Zeiss). A 1000 μm-thick virtual straight line was drawn on images at the midline to separate the hemispheres. Numbers of axons crossing the line were counted blindly and averages for each of the three groups were calculated.

### Statistical Analysis

Statistical analyses were performed using SPSS (version 15, SPSS, Inc.) software. Data were evaluated by one-way ANOVA followed by LSD tests. Data are presented as mean ± SD values. Throughout the study, *p*-values < 0.05 were considered significant.

## Results

### High-Frequency rTMS Decreases Infarct Volume and Enhances Microcirculation

**FIGURE 2:**
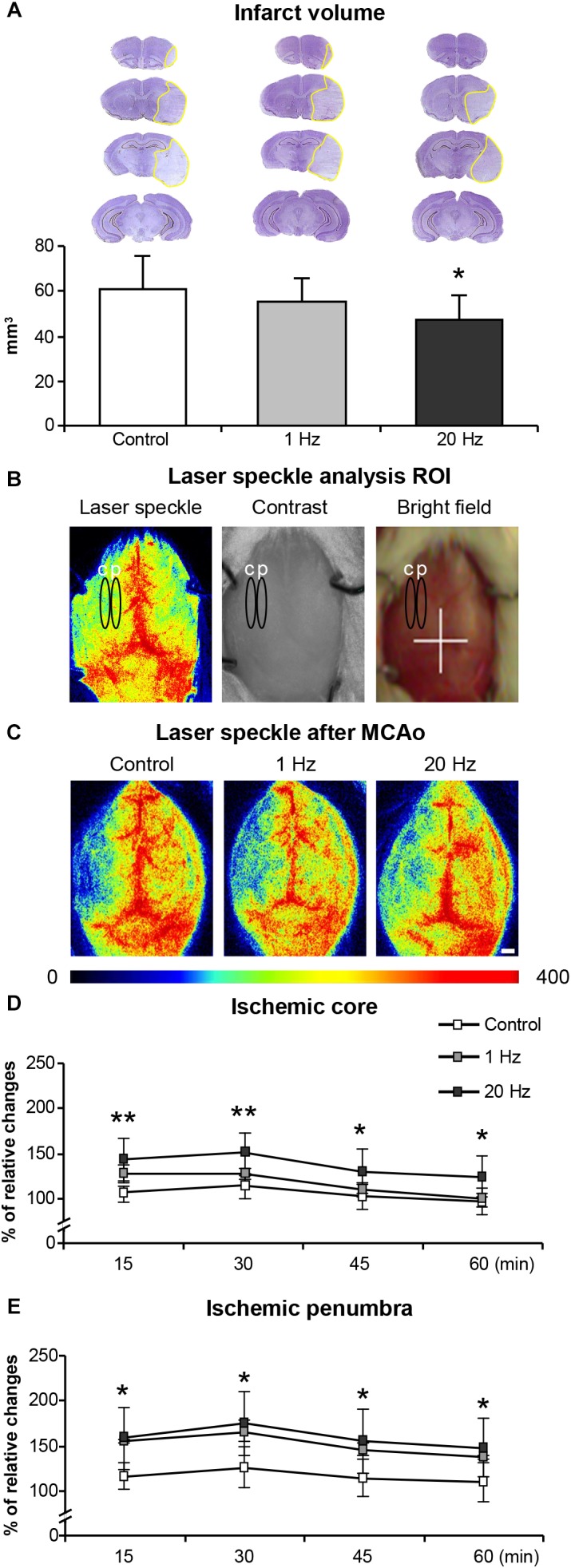
Effects of rTMS on infarct volume and post-ischemic regional cerebral blood flow (CBF). Infarct volume **(A)**, laser speckle analysis ROI **(B)**, laser speckle images (LSI) after rTMS treatment **(C)** microcirculation on ischemic core **(D)**, and penumbra **(E)**. Letter c on **(B)** represents ischemic core area, letter p represents penumbra area. Data were evaluated by one-way ANOVA followed by LSD tests. Data are presented as mean ± SD values (*n* = 8–9 mice/group for infarct volume/*n* = 5/group for microcirculation analysis). ^∗^*p* < 0.05 compared with control for **(A)**, ^∗∗^*p* < 0.01/^∗^*p* < 0.05 comparison between control and 20 Hz rTMS groups for **(D,E)**.

The LDF values reproducibly decreased to -15–20% of pre-ischemic control during MCAo. Reproducibility of reperfusion onset was also recorded by LDF after 90 min of MCAo. LDF measurements during ischemia did not reveal differences in CBF differences among the experimental groups (data not shown). Infarct volume as evaluated by cresyl violet staining was significantly reduced in the high-frequency rTMS applied animals when compared with control or low-frequency groups (Figure 2A). Interestingly, low-frequency rTMS also resulted in a slight, but not significant, decrease in the infarct volume. Moreover, to investigate the hemodynamic effects of rTMS, instantaneous CBF of the ischemic core was evaluated at different time points using LSI (Figure 2C).

Although we used LDF for the evaluation of CBF changes, it does not provide accurate absolute regional CBF values because of the small size of flexible optic probe used. Therefore, we have evaluated regional CBF by using more accurate LSI just after rTMS treatments represented in Figure 2B,C. In contrast to control and low-frequency TMS, high-frequency rTMS significantly increased regional CBF above the ischemic core and penumbra at 15, 30, 45, and 60 min after reperfusion (Figure 2D,E). More pronounced increase in CBF was observed in the ischemic core region in high-frequency rTMS treated animals but not in low-frequency group (Figure 2C). Low-frequency rTMS increased CBF particularly in the ischemic penumbra region but it was not statistically significant (Figure 2E).

### High-Frequency rTMS Reduces Apoptotic Cell Death and Capillary Injury, While Promoting Neuronal Survival

**FIGURE 3:**
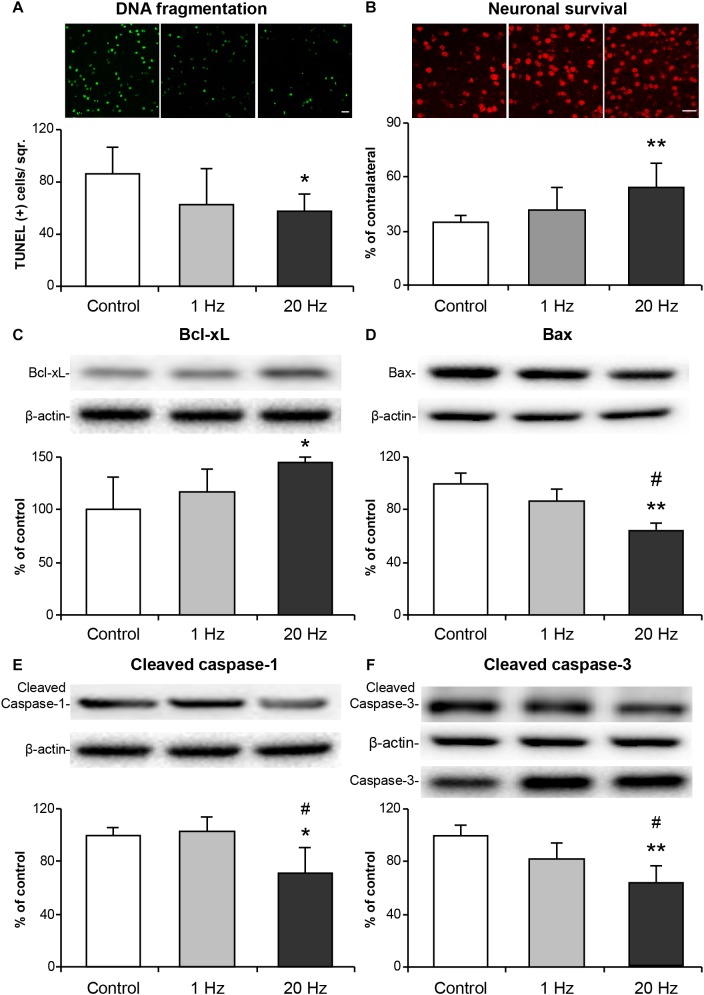
20 Hz rTMS reduces DNA fragmentation, pro-apoptotic protein expressions and promotes neuronal survival. Disseminate neuronal injury in the striatum was assessed by terminal transferase dUTP nick end labeling (TUNEL) **(A)**, neuronal survival was assessed using NeuN stained brain sections **(B)**, expressions of anti-apoptotic Bcl-xL **(C)**, pro-apoptotic Bax **(D)**, inflammation related cleaved caspase-1 **(E)**, pro-apoptotic cleaved caspase-3 **(F)** were demonstrated with Western blot. Data are mean ± SD values (*n* = 6–7 mice/group). Data were evaluated by one-way ANOVA followed by LSD tests. Data are presented as mean ± SD values. ^∗∗^*p* < 0.01/^∗^*p* < 0.05 compared with control/^#^*p* < 0.05 compared with 1 Hz rTMS. Scale bars are 100 μm.

To determine the effects of rTMS on apoptotic cell death mechanisms, the second experimental setup which consisted of 30 min of MCAo followed by 72 h reperfusion was used, as it induces a less severe injury which is accompanied by selective neuronal cell death (Beker et al., 2015). DNA fragmentation as assessed at the end of 72 h using TUNEL assay was significantly decreased in the high-frequency rTMS group (Figure 3A). Neuronal survival, as evaluated by NeuN immunopositive cell count in the ipsilateral and contralateral striata and given as the per cent of total NeuN+ cells of the contralateral hemisphere, was also significantly increased in the 20 Hz rTMS group (Figure 3B). On the other hand, low-frequency rTMS demonstrated a slight (and non-significant) decrease in DNA fragmentation and a slight increase in the neuronal survival when compared with control. We have also studied capillary injury after 30 min of MCAo by evaluating CD31 positive cells. Vascular density was significantly increased in 20 Hz rTMS treated animals compared with controls and 1 Hz rTMS groups. As compared with control, 20 Hz rTMS protected capillary integrity significantly (*p* = 0.007). Capillary density were 85 ± 8 in 20 Hz rTMS, 67 ± 6 in 1 Hz rTMS (*p* = 0.008 compared with 20 Hz rTMS) and 65 ± 7 vessel/square in control animals (*p* = 0.007 compared with 20 Hz rTMS). Next, we investigated the expressions of apoptosis-related proteins; Bcl-xL, Bax, cleaved caspase-1, and cleaved caspase-3 in the ipsilateral hemisphere using Western blot analysis. High-frequency rTMS significantly increased protein levels of anti-apoptotic Bcl-xL (Figure 3C), while decreasing protein levels of neuronal death-associated Bax (Figure 3D), cleaved caspase-1 (Figure 3E) and cleaved caspase-3/caspase-3 (Figure 3F).

### rTMS Reduces Inflammatory Responses, Increases Angiogenesis, Growth Factors, and Reduces Axonal Outgrowth Inhibitors Related Gene Expressions

**FIGURE 4:**
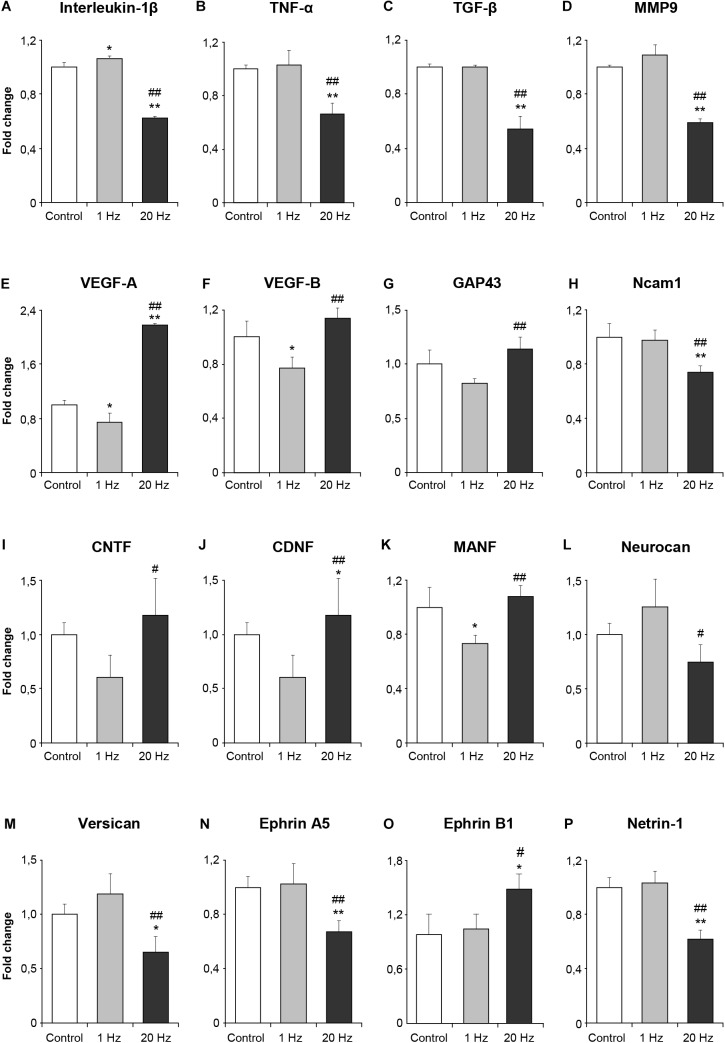
Effect of rTMS on gene expression after cerebral ischemia. Inflammation-related genes after ischemia IL1β **(A)**, TNFα **(B)** and TGFβ **(C)** and MMP9 **(D)**, angiogenesis-related VEGF-A **(E)** and VEGF-B **(F)**, neuroplasticity related GAP43 **(G)** and Ncam1 **(H)**, the trophic factors CNTF **(I)**, CDNF **(J)**, MANF **(K)** and Neurocan **(L)**, growth inhibiting genes Versican **(M)**, Ephrin A5 **(N)** and Ephrin B1 **(O)**, axonal growth related gene Netrin1 **(P)**. Data were evaluated by one-way ANOVA followed by LSD tests. Data are presented as mean ± SD values (*n* = 8–9 mice/group). ^∗∗^*p* < 0.01/^∗^*p* < 0.05 compared with control, ^##^*p* < 0.01/^#^*p* < 0.05 compared with 1 Hz rTMS.

The sustained effect of rTMS on the neurons was ascribed to its ability to modulate gene expression and hence, cause long-term alterations in the intracellular signaling. To study whether low-frequency and high-frequency rTMS exerted such effects, we determined the expressions of a number of genes related to inflammation, angiogenesis, plasticity and trophic factors in animals subjected to no stimulation, 1 Hz rTMS or 20 Hz rTMS after 90 min MCAo using qPCR analyses. High-frequency rTMS significantly decreased the expressions of inflammation-related genes; IL1β, TNFα, and TGFβ (Figure 4A–C) and MMP9 (Figure 4D), while significantly upregulating angiogenesis-related VEGF-A and VEGF-B (Figure 4E,F). Evaluation of neuroplasticity related gene expression demonstrated that high-frequency rTMS significantly increased growth-promoting gene GAP43 (Figure 4G), whereas significantly decreased growth inhibiting genes Ncam1 (Figure 4H), Neurocan, Versican, Ephrin A5, and Ephrin B1 (Figure 4L–O) when compared with low frequency rTMS. Of the trophic factors analyzed, high-frequency rTMS significantly increased the expression of CNTF, CDNF, and MANF (Figure 4I–K) when compared with low frequency rTMS. It is interesting that the expression of axonal growth related gene Netrin1 was downregulated by 20 Hz rTMS (Figure 4P).

### Functional Recovery Is Enhanced by High-Frequency rTMS

**FIGURE 5:**
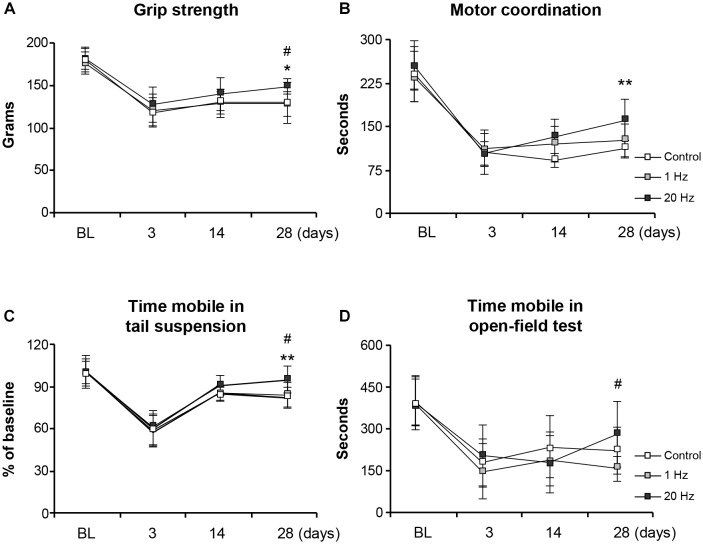
20 Hz rTMS promotes post-ischemic grip strength and motor coordination, thus reducing depression. Evaluation of post-ischemic recovery of grip strength **(A)**, motor coordination **(B)**, depression **(C)**, and spontaneous activity **(D)**. Data were evaluated by one-way ANOVA followed by LSD tests. Data are presented as mean ± SD values (*n* = 12 mice/group). **p < 0.01/*p < 0.05 compared with control, ^#^*p* < 0.05 compared with 1 Hz rTMS.

Functional recovery was assessed at days 3, 14, and 28 in the third experimental setup in which mice were subjected to 30 min MCAo. Grip strength and motor coordination assessments indicated a significant increase in the motor force in the paretic right forelimb in the high-frequency rTMS group when compared with control animals at 28 days after MCAo (Figure 5A,B). Time spent mobile in tail suspension test was evaluated to investigate the effect of rTMS on depression. Even though there was no significant difference among the groups at day 3 or day 14, high-frequency rTMS significantly increased time spent mobile at day 28 when compared with control and low-frequency rTMS (Figure 5C). High-frequency rTMS also significantly increased the spontaneous exploration behavior, as evaluated by open-field test, at day 28 when compared with low-frequency rTMS (Figure 5D). As expected, high-frequency rTMS significantly improved all the functional parameters evaluated, supporting the human studies indicating its promising effects on motor functions (Takeuchi et al., 2009; Khedr et al., 2010).

### High-Frequency rTMS Decreases Striatal Atrophy and Glial Scar Formation, While Promoting Neurogenesis, and Axonal Projections

**FIGURE 6:**
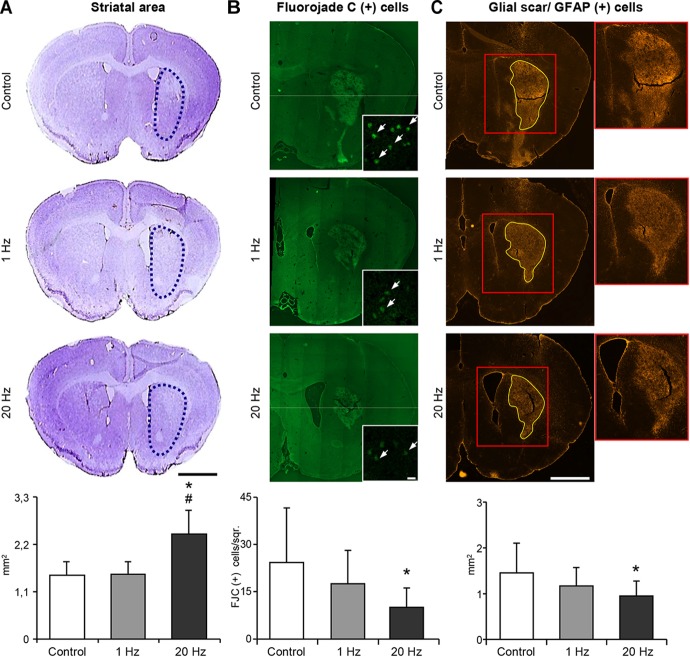
20 Hz rTMS inhibits delayed neuronal loss and reduces glial scar formation. Striatal atrophy was assessed by cresyl violet staining in the ischemic striatum of animals subjected to 30 min ischemia followed by 42 days reperfusion **(A)**. Dotted black lines indicate the area of ischemic striatum in cresyl violet stained sections. Quantification of striatal area is given in the graphs below. Significantly increased striatal area indicates that 20 Hz rTMS significantly decreased striatal atrophy. Degenerating cells were counted using Fluorojade C stained brain sections **(B)**. 20 Hz rTMS-applied animals displayed significantly decreased number of Fluorojade C (+) cells. White arrows indicate Fluorojade C (+) cells. Glial scar area was measured in GFAP-stained sections in animals subjected to 30 min ischemia followed by 42 days reperfusion **(C)** and high-frequency rTMS resulted in the significantly decreased glial scar area when compared with control. Data were evaluated by one-way ANOVA followed by LSD tests. Data are presented as mean ± SD values (*n* = 7–8 mice/group). ^∗^*p* < 0.05 compared with control group and ^#^*p* < 0.05 compared with 1 Hz rTMS. Scale bar is 2 mm **(A,C)** 50 μm **(B)**.

**FIGURE 7:**
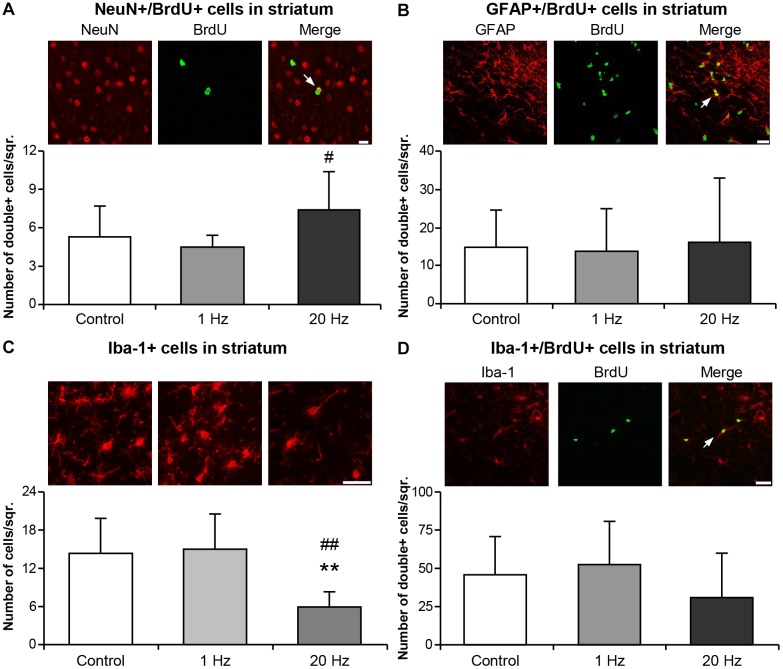
Neurogenesis was induced and Iba-1+ microglial activation was inhibited by 20 Hz rTMS. Post-ischemic cell proliferation was assessed by 5-Bromo-2′-deoxyuridine (BrdU) immunostaining in the ischemic striatum. Co-expressions of BrdU and NeuN **(A)**, GFAP **(B)**, and Iba-1 **(D)**, and microglial activation **(C)** were evaluated from animals subjected to 30 min ischemia followed by 42 days reperfusion. Data were evaluated by one-way ANOVA followed by LSD tests. Data are presented as mean ± SD values (*n* = 7–8 mice/group). ^∗∗^*p* < 0.01 compared with control group and ^##^*p* < 0.01/^#^*p* < 0.05 compared with 1 Hz rTMS. White arrows indicate double immunopositive stainings. Scale bar is 100 μm **(A,D)** 50 μm **(B,C)**.

Striatal area was evaluated to examine the effect of rTMS treatments on long term tissue damage. Striatal area (Figure 6A), number of degenerating cells (Figure 6B) and glial scar area (Figure 6C) were significantly decreased in the 20 Hz rTMS group. To examine the proliferating cells in the ischemic striatum, BrdU+ cells were analyzed and results indicated a significant increase in the number of BrdU+ cells in animals of the third experimental setup. When stained with specific cellular markers, BrdU+ cells indicate the differentiation of the specific cell type. Thus, double staining with mature neuronal nuclei, NeuN, and BrdU antibodies revealed significantly increased neurogenesis in the high-frequency rTMS group when compared with low-frequency rTMS (Figure 7A). Number of proliferating astrocytes, as demonstrated by double immunostaining with glial marker, GFAP, and BrdU was slightly, but not significantly, increased with 20 Hz rTMS (Figure 7B). We further analyzed the microglial activation in the ischemic striatum by counting Iba1+ cells and found that the number of Iba1+ cells in ischemic striatum was significantly reduced by high-frequency rTMS (Figure 7C). On the other hand, high-frequency rTMS slightly decreased the number of Iba1/BrdU double immunopositive cells in the ischemic striatum; while low-frequency rTMS resulted in a slight increase (Figure 7D).

**FIGURE 8:**
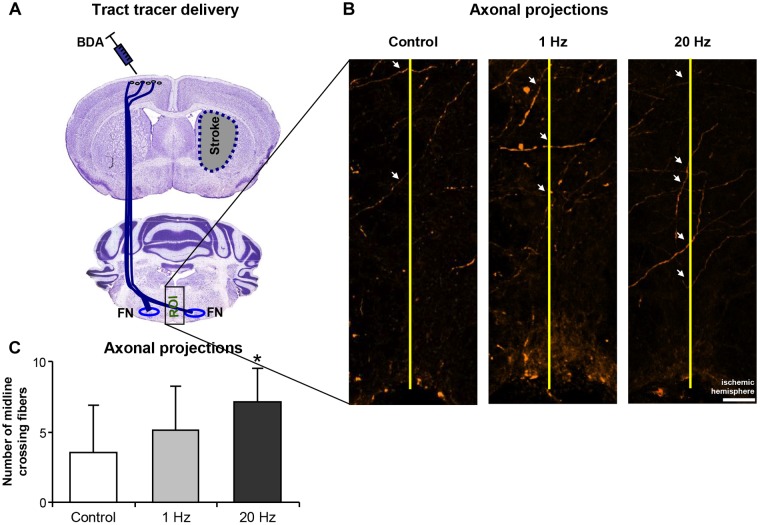
20 Hz rTMS stimulates plasticity following cerebral ischemia. Midline crossing axon fibers in facial nuclei was traced by BDA administration after cerebral ischemia (for placement of tracer injections see **A**). Axonal projections were evaluated by drawing a virtual straight line (yellow line, **B**) at the midline to separate the two hemispheres and to count the number of axons crossing the virtual line **(C)**. Data were evaluated by one-way ANOVA followed by LSD tests. Data are presented as mean ± SD values. ^∗^*p* < 0.05 compared with control group. Arrows show axonal projections. Scale bar is 100 μm.

In addition, in the third experimental setup, corticobulbar projections following ischemia were determined using BDA injections after 28 days of daily rTMS treatment to allow the completion of the formation of new axonal connections (Figure 8A). Axonal projections were evaluated at the facial nucleus level (bregma -5.8 to -6.3 mm) by drawing a virtual straight line (yellow line, Figure 8B) at the midline to separate the two hemispheres and to count the number of axons crossing the virtual line. Significantly increased number of axonal projections was observed in the 20 Hz rTMS group, whereas 1 Hz rTMS only slightly increased the number of fibers crossing to the other hemisphere (Figure 8C).

## Discussion

According to the World Health Organization, stroke is the third leading cause of disability worldwide (Johnson et al., 2016). Although it is estimated that each year 15 million people suffer from a stroke episode, due to the limited usable time frame of tissue plasminogen activator, only a small per cent of patients can actually benefit from the only treatment available (Thrift et al., 2014). To this end, rTMS was promoted as a non-invasive approach that can help restoring the unbalanced interhemispheric inhibition which results in increased inhibition in the ischemic hemisphere and subsequent worsening in motor function. The physiological effect of rTMS on neuronal activity has not been well-studied. However, the electric currents necessary to stimulate the brain can be produced by rapidly changing magnetic fields. It is assumed that the mechanisms underlying rTMS after-effects appear as observed for long-term potentiation (LTP) and long-term depression (LTD) (Klomjai et al., 2015).

The effects of rTMS on motor recovery following stroke have been reported in meta-analyses of human studies with 362 patients (Hsu et al., 2012; Le et al., 2014). However, the cellular mechanisms mediating rTMS’s neuroprotective and restorative activities need to be elucidated. For these aims we made use of mice to examine the roles of low- and high-frequencies of rTMS in the development of (i) brain injury; (ii) brain perfusion; (iii) apoptosis and related signaling pathways; (iv) inflammation, angiogenesis, growth factors and axonal outgrowth related gene expressions after 30 and 90 min of FCI. Thirty min of FCI is a short term ischemic injury model based on MCAo, causing only disseminate neuronal injury in the ischemia-vulnerable striatum, but not the overlying cortex. In this model, acute pathophysiological changes are completed 72 h after MCAo (Kilic et al., 2013). However, 90 min of MCAo causes necrotic cell death or infarct development in the striatum and overlying cortex (Kilic et al., 2013). Therefore two different durations of MCAo model were recruited for the evaluation of acute role of rTMS in the development of injury and related events. For the post-acute role of rTMS, 30 min of MCAo and 42 days reperfusion model was used. For the evaluation of restorative effects of rTMS, treatment was started 3 days after MCAo, just after the completion of acute pathophysiological changes, in order to investigate spontaneous locomotor activity, perilesional tissue remodeling, axonal sprouting of corticobulbar tracts, glial scar formation, and neurogenesis. Here, we have shown that high-frequency rTMS reduced infarct volume, apoptosis and inflammation-related gene expression and increased neuronal survival, neurogenesis, contralesional axonal projections and regional CBF, which resulted in improved functional recovery.

For the stimulation of brain, we chose figure-of-eight coiled rTMS due to its producing maximum current at the midline of the figure-eight (Hallett, 2007). Although we assumed that both hemispheres might have been affected by rTMS stimulation to some extent, the main stimulation site was set as the primary motor cortex (left M1). Maximum current or MEPs were measured at the biceps femoris (BF) muscle of the right hind limb using an electromyography as previously described (Ogiue-Ikeda et al., 2003; Yoon et al., 2011). Brain perfusion was monitored by LDF. Although LDF has been in use for the analyses of CBF changes after ischemic stroke in animal experiments, it does not provide accurate absolute regional CBF values because of the small size of flexible optic probe used. Therefore, we have evaluated real time regional CBF also by using more accurate LSI method just after rTMS treatments (Beker et al., 2015). Our data suggest that particularly high- frequency rTMS increased regional CBF in the ischemic core and penumbra. Low-frequency rTMS had almost no effect on brain perfusion in the ischemic-core, while moderate increase in CBF in penumbra was observed. To the best of our knowledge, there is no study measuring CBF in mice reported. However, an overall increase in CBF was observed after 15 Hz rTMS (Loo et al., 2003) and also partially increased CBF on the ipsilateral side of the stimulation after 1 Hz low-frequency of rTMS treatment was revealed in humans (Mesquita et al., 2013).

In the present study, we found that 20 Hz rTMS reduced infarct volume and apoptotic cell death while enhancing neuronal survival, possibly through the enhancement of excitability of neurons in the ipsilateral hemisphere and regulation of apoptosis-related proteins Bax, Bcl-xL, caspase 1, and caspase 3. Moreover, in the subacute phase high-frequency rTMS inhibited delayed neuronal loss and glial scar formation after cerebral ischemia. Increased number of surviving neurons and decreased DNA fragmentation as well as striatal atrophy significantly correlated with functional recovery on the behavioral tests performed. In accordance with our results, high-frequency rTMS was reported to reduce ischemic injury, analyzed by microPET 7 days after MCAo in rats (Gao et al., 2010), and improves functional recovery and cell proliferation 14 days after MCAo (Luo et al., 2017). Furthermore, it was also revealed that high frequency rTMS increased cell proliferation and synaptic plasticity and inhibited apoptotic cell death through activation of BDNF, CREB, ERK, and AKT signaling pathways in neuronal cell culture after oxygen glucose deprivation (Baek et al., 2018). Beneficial effects of rTMS on motor recovery were reported in stroke patients (Hsu et al., 2012). Indeed, we showed that muscular strength and motor coordination were enhanced by high-frequency rTMS. Depression analysis of post-ischemic animals revealed that high-frequency rTMS reduced the depression levels of animals. The ameliorating effect of rTMS on depression has long been known and to date, there are at least four FDA approved rTMS systems for clinical use in depression (Zhang et al., 2014).

Focal cerebral ischemia has been reported to induce neurogenesis in the injured hemisphere in humans (Huttner et al., 2014). In addition to the subventricular zone and subgranular zone of the dentate gyrus in which new neurons are produced in the adult brain, neurogenesis also occurs in the ischemic striatum or cortex, although these areas are not normally accepted as neuron-generating areas in the adult brain (Lindvall and Kokaia, 2015). It is believed that enhancing the endogenous neurogenesis after stroke may support the tissue repair and functional recovery (Lu et al., 2017). Here, we demonstrated that 20 Hz rTMS induced neurogenesis in the ischemic striatum 42 days after MCAo, suggesting that high-frequency rTMS can be implemented to promote functional recovery. In line with our findings, rTMS-induced neurogenesis was observed in the hippocampi of healthy (Ueyama et al., 2011) and ischemic rats (Guo et al., 2014, 2017; Luo et al., 2017). In addition to neurogenesis, astrocyte and microglial activation also play a role in the repair and plasticity processes in response to ischemic stroke. Consistent with our results, proliferating astrocytes were evaluated in rat hippocampus after high intensity TMS application and a significant alteration was not found (Zhang et al., 2014; Luo et al., 2017). Therefore, we surmised that the increase in neurogenesis may result from the rTMS-induced neural stem cell proliferation and subsequent preferential differentiation into neuronal lineage rather than glial cells. The number of Iba1+ cells was significantly reduced with 20 Hz rTMS in the ischemic striatum and we observed a non-significant trend toward reduction in new-born microglia (BrdU/Iba1 double immunopositive cells). Similarly, decreased number of GFAP and Iba1+ cells was shown in a rat model of spinal cord injury in response to high-frequency (25 Hz) rTMS treatment (Kim et al., 2013) as well as in rats treated with rTMS after focal brain injury (Sasso et al., 2016).

Prolonged aftereffects imply that high-frequency rTMS may result in changes in the overall gene expression profile. Indeed, we observed significant changes in a number of genes related with inflammation, synaptic plasticity, trophic factors, and angiogenesis in the high-frequency rTMS group. Our results indicate that rTMS reduces inflammation-related gene expression (IL1β, TNFα, MMP9, and TGFβ), as suggested using a pulsed electromagnetic field following ischemia (Pena-Philippides et al., 2014). Therefore, we hypothesize that high-frequency rTMS alleviates inflammatory response to ischemic injury not only by downregulating the inflammation-related genes but also by inhibiting the accumulation of Iba1+ cells in the ischemic striatum.

In addition, angiogenesis-related gene expression (VEGF-A, VEGF-B) was induced with high-frequency rTMS. It is of importance that VEGF-A and VEGF-B were also implicated with neurogenesis (Jin et al., 2002; Sun et al., 2006), further supporting the use of rTMS to stimulate neurogenesis following ischemic injury. Moreover, expressions of neurotrophic factors were significantly upregulated by high-frequency rTMS, suggesting that these factors may be important in the therapeutical activity of rTMS.

In parallel with gene expression data demonstrating the downregulation of axon growth inhibitor genes by high-frequency rTMS, we observed that contralesional axonal projections were at least doubled by high-frequency rTMS. Consistent with previous reports suggesting rTMS-induced plasticity (Ogiue-Ikeda et al., 2003; Ahmed and Wieraszko, 2006), we demonstrated neural plasticity in both molecular and structural levels and connected the increased plasticity related molecules reported in rodent studies to the motor functional recovery observed in human patients.

Notably, there are also discrepancies in the clinical data on the outcomes of low-frequency and high-frequency rTMS; however, high-frequency rTMS is generally more likely to produce favorable results. Overall, our results demonstrated that high-frequency (20 Hz) rTMS resulted in better outcomes than low-frequency (1 Hz) rTMS in both acute and subacute ischemic injury models in mice.

## Conclusion

Our results demonstrate that high-frequency rTMS decreases infarct volume and apoptosis, activates neuronal survival, neurogenesis, neuronal plasticity, and regional CBF. In addition, rTMS induces changes in gene expression, axonal projections and eventually functional recovery, altogether suggesting the complex nature of rTMS-induced mechanisms. Although rTMS was believed to exert its effects mainly by blocking apoptosis, we propose a wider range of mechanisms involved in its favorable effects, mainly consisting of neural-related processes. Overall, our data strongly support the rationale for the use of non-invasive high-frequency rTMS therapy in stroke patients in order to promote functional recovery through the induction of endogenous repair and recovery mechanisms of the brain.

## Ethics Statement

All experimental procedures were carried out with government approval according to NIH guidelines for the care and use of laboratory animals. Ethical committee approval was obtained from Istanbul Medipol University.

## Author Contributions

ABC, MB, and EK: study concept and design. ABC, MB, BC, EY, and AC: data acquisition and analysis. BY, LH, SK, DH, TD, and EK: manuscript and figure drafting.

## Conflict of Interest Statement

The authors declare that the research was conducted in the absence of any commercial or financial relationships that could be construed as a potential conflict of interest.

## Supplementary Material

TABLE S1 List of qPCR primers used. Primer sequences were obtained from Harvard Primerbank.
