# Supplementary figures and images for: Acute and Post-acute Neuromodulation Induces Stroke Recovery by Promoting Survival Signaling, Neurogenesis, and Pyramidal Tract Plasticity
Source: Front Cell Neurosci. 2019 Apr 12;13:144. doi: 10.3389/fncel.2019.00144 (PMC6474396; doi:10.3389/fncel.2019.00144)

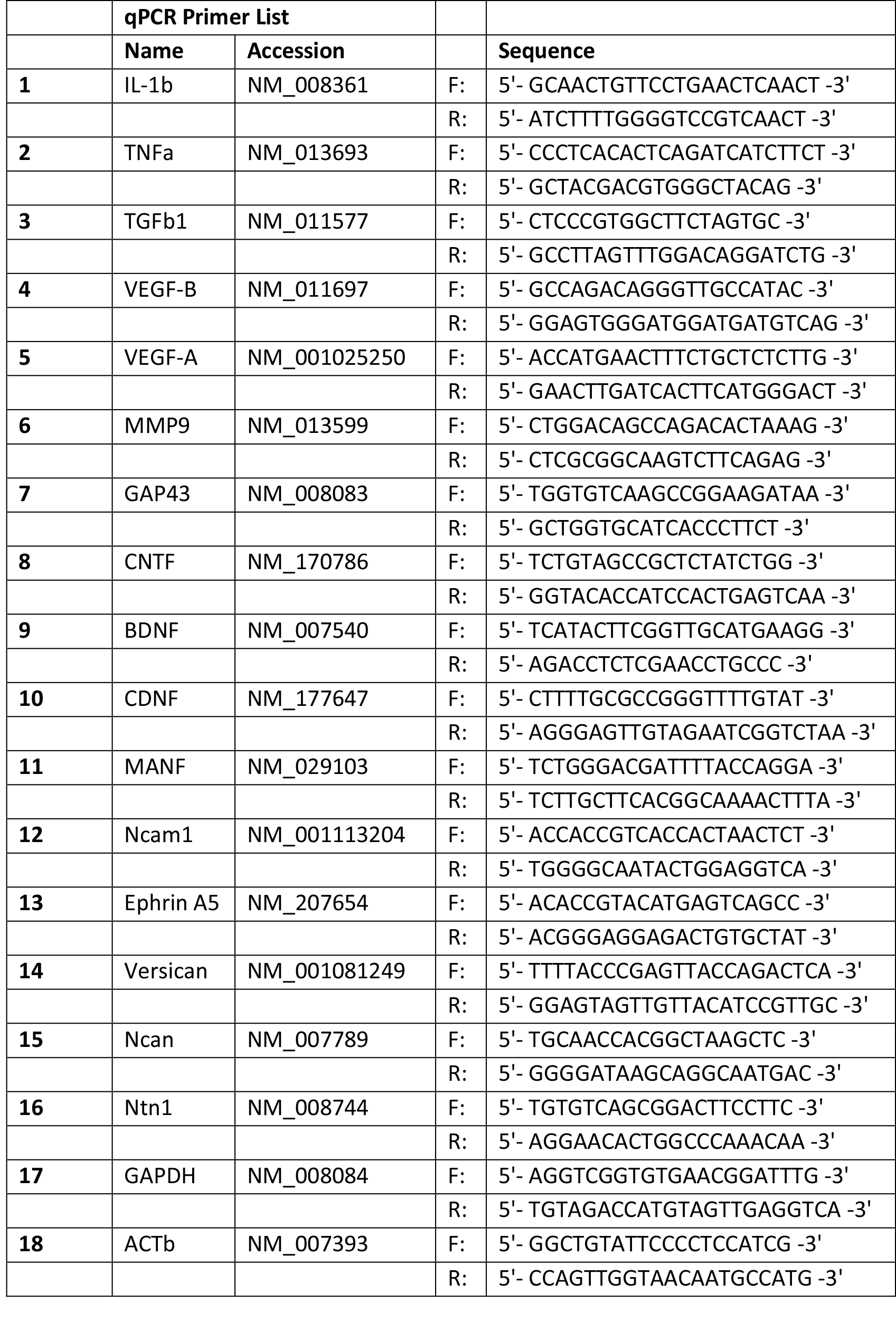

Supplement: TABLE S1 — List of qPCR primers used. Primer sequences were obtained from Harvard Primerbank. [file Table_1.TIF]
